# Investigating the Allosteric Regulation of YfiN from *Pseudomonas aeruginosa*: Clues from the Structure of the Catalytic Domain

**DOI:** 10.1371/journal.pone.0081324

**Published:** 2013-11-22

**Authors:** Giorgio Giardina, Alessandro Paiardini, Silvia Fernicola, Stefano Franceschini, Serena Rinaldo, Valentina Stelitano, Francesca Cutruzzolà

**Affiliations:** 1 Department of Biochemical Sciences, Sapienza University of Rome, Rome, Italy; 2 Istituto Pasteur - Fondazione Cenci Bolognetti, Department of Biochemical Sciences, Sapienza University of Rome, Rome, Italy; University of Canterbury, New Zealand

## Abstract

*Pseudomonas aeruginosa* is responsible for a plethora of biofilm mediated chronic infections among which cystic fibrosis pneumonia is the most frightening. The long-term survival strategy of *P. aeruginosa* in the patients lungs is based on a fine balance of virulence *vs* dormant states and on genetic adaptation, in order to select persistent phenotypes as the small colony variants (SCVs), which strongly correlate with antibiotic resistance and poor lung function. Recent studies have coupled SCV with increased levels of the signaling molecule cyclic di-GMP, and demonstrated the central role of the diguanylate cyclase YfiN, part of the tripartite signaling module YifBNR, in c-di-GMP dependent SCV regulation. YfiN, also called TpbB, is a multi-domain membrane enzyme connecting periplasmic stimuli to cytosolic c-di-GMP production by an allosteric inside-out signaling mechanism that, due to the lack of structural data, is still largely hypothetical. We have solved the crystal structure of the catalytic domain (GGDEF), and measured the enzymatic activity of the cytosolic portion in real-time by means of a newly developed method. Based on these results we demonstrate that, unlike other diguanylate cyclase, YfiN does not undergo product feedback inhibition, and that the presence of the HAMP domain is required for dimerization and catalysis. Coupling our structural and kinetic data with an *in silico* study we are now able to propose a model for the allosteric regulation of YfiN.

## Introduction

The majority of chronic infections involve a biofilm stage. In most bacteria, the synthesis of the ubiquitous second messenger cyclic di-GMP (c-di-GMP) represents a common principle in the formation of otherwise highly diverse and species-specific biofilms [1–4]. Therefore, c-di-GMP signaling pathways play a key role in chronic infections [4]. The human pathogen *Pseudomonas aeruginosa* is responsible for a plethora of biofilm-mediated chronic infections among which cystic fibrosis (CF) pneumonia is the most frightening [5]. During long-term colonization of CF lungs *P. aeruginosa* undergoes specific genotypic adaptation to the host environment and, after a yearlong persistence, it develops small-colony variants (SCVs) [6–8]. SCVs, which display high intracellular c-di-GMP levels [9–11], are characterized by enhanced biofilm formation, high fimbrial expression, repression of flagellar genes, resistance to phagocytosis, and enhanced antibiotic resistance [10–14]; their appearance correlates with a poor patient clinical outcome [6,12,15]. A direct relationship between the presence of bacterial persister cells and the recalcitrant nature of chronic infections has been proposed [16].

The c-di-GMP metabolism in *P. aeruginosa* is highly complex: 42 genes containing putative diguanylate cyclases (DGCs) and/or phosphodiesterase are present [17]. It has been shown that SCVs generated *in vitro* as well as obtained from clinical isolates contain mutations that upregulate the activity of a specific DGC, i.e. YfiN (also called TpbB [18], encoded by the PA1120 gene), suggesting a key role of this enzyme. Since YfiN is the effector protein of a tripartite signaling module YifBNR [14,19,20], in this work we choose to use the name YfiN for coherence with the other two members of the operon *PA1119* and *PA1121*, which, in the Pseudomonas genome database (http://www.pseudomonas.com/), are called YfiB and YfiR, respectively. Formation of SCVs depends on enhanced c-di-GMP output by YfiN, which elevates transcription of the *pel* operon [11,14,21]. The YfiBNR system likely contributes to the degree of persistence of *P. aeruginosa* cells in CF lungs. Jenal and coworkers [20], have shown, by looking at mutations in the YfiBNR genes found in clinical strains of *P. aeruginosa*, that the activity of YfiN (and the occurrence of the SCV phenotype) is under continuous cycles of positive and negative selection; the same group proposed that this mechanism may contribute to the *in vivo* fitness of *P. aeruginosa* during chronic lung infections.

**Figure 1:**
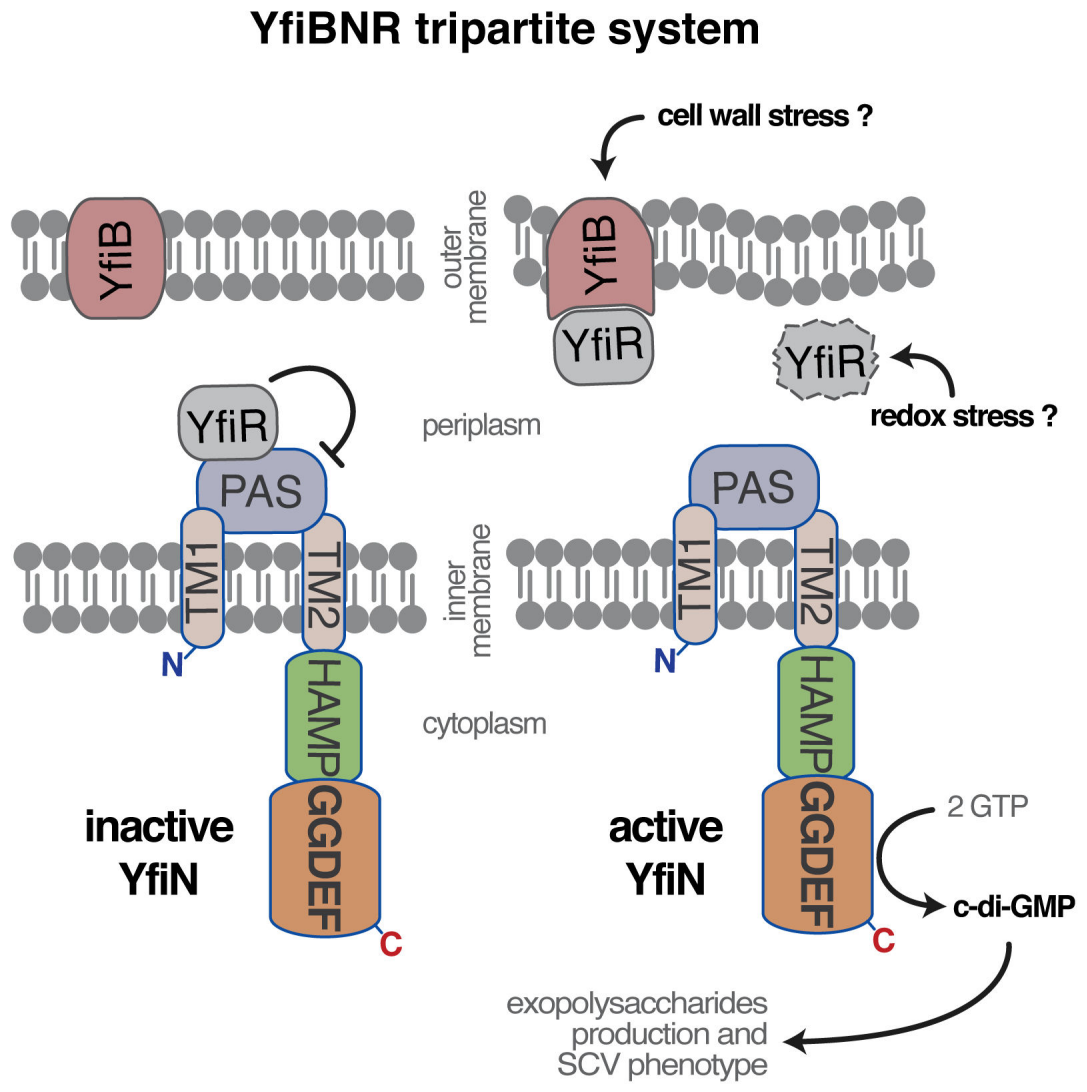
YfiBNR tripartite system organization. Schematic representation of the localization the YfiBNR system. YfiN is repressed by the specific interaction of YfiR with its periplasmatic domain, while dissociation of the complex, and the consequent activation of YfiN, may be induced by a YfiB-mediated cell wall stress sensing mechanism and/or by redox driven misfolding of YfiR [20].

Figure 1 illustrates the composition of the YfiBNR system. YfiN is an inner membrane protein composed of three domains: a periplasmic PAS domain, two transmembrane helices and a cytosolic portion of the protein, which includes an HAMP domain and a cyclase domain (named GGDEF from the conserved residues in the active site). The negative regulator YfiR [14,20] is a dimeric periplasmic protein which controls the activity of YfiN by binding to the PAS domain of the DGC. YfiR has also been proposed to sense the redox state (and therefore oxygen levels) thus possibly (and intriguingly) conveying signals related to the switch to the anaerobic mode of growth (including denitrification [22]), typical of *P. aeruginosa* chronic infections. A third component of the system is the YfiB protein, spanning the outer membrane and the peptidoglycan and involved in binding YfiR, thus relieving the repression of YfiN activity (Figure 1). No structural data are available for this system and therefore several aspects of this signaling pathway are yet to be discovered in order to define its role in SCV formation during chronic infections.

We have solved the crystal structure of the cyclase domain (GGDEF) of YfiN, completed its multi-domain structure by homology modeling, and performed a biochemical characterization of two constructs of the cytoplasmic portion of YfiN. We also measured the enzymatic activity by using a new approach for c-di-GMP detection in real-time [23]. Here we show that, unlike other DGC enzymes, YfiN does not undergo product feedback inhibition, and that the *in vitro* activity depends on the presence of the HAMP domain. Moreover, we propose that the predicted PAS domain is more likely to fold as the periplasmic N-terminal domain of the receptor LapD from *P. fluorescens* [24]. Coupling structural and biochemical data, we are able to suggest a mechanistic model for the allosteric regulation of YfiN in response to YfiR binding.

## Results and Discussion

### Crystal structure of the GGDEF domain

Based on fold and secondary structure prediction [25,26], YfiN is organized in three domains: a N-terminal domain, spanning residues 35-161, delimited by two transmembrane helices (TM1: residues 14-34 and TM2: 162-182); a HAMP domain (residues 183-246); a C-terminal GGDEF domain (residues 249-406). In order to gain structural insights on the allosteric regulation of YfiN, we expressed the cytosolic portion of the protein, including the HAMP and the GGDEF cyclase domain (YfiN_HAMP-GGDEF_; residues 183-435). This truncated construct resulted monomeric in solution. After extensive crystallization trials we could finally collect a complete data set at 2.8 Å resolution from one single hexagonal crystal. The crystal belonged to the P6_5_22 space group. Surprisingly, analysis of the unit cell solvent content (Matthews coefficient) clearly indicated that only one of the two domains of the protein could be physically present in the crystal lattice since fitting both domains in the cell volume would result in a solvent content of 11%, which is too low for a protein crystal. The solved structure confirmed that YfiN_HAMP-GGDEF_ had actually undergone proteolysis and that only the GGDEF domain had crystallized (YfiN_GGDEF_). The quality of the diffraction data is good and electron density is clearly visible for all main chain atoms spanning from residue 254 to 414 of the GGDEF domain (Figure S1 and Table 1).

**Table 1:** Data collection and refinement statistics for YfiN_GGDEF_.

**Coordinates**	4IOB
**Data collection**	
Beamline	ESRF (ID14-1)
Space group	P 6_5_ 2 2
Cell dimensions	
*a* = *b*, *c* (Å)	70.35, 106.87
Resolution (Å)	40.0-2.78 (2.94-2.78)
*R*_factor_	8.3 (68.2)
*I* / sigma *I*	31.1 (3.3)
Completeness (%)	99.6 (98.2)
Reflections	
Observed	59914 (6510)
Unique	4343 (666)
B Wilson	57.9
**Refinement**	
Resolution (Å)	40.17-2.78
No. unique reflections	4095
*R*_work_ / *R*_free_	27.8 / 28.0
Mean *B*-factor (Å^2^) (atoms)	
Protein	48.1 (1235)
tert-butanol	33.3 (5)
Glycerol	54.9 (6)
R.m.s. deviations	
Bond lengths (Å)	0.0014
Bond angles (°)	0.460
Ramachandran: (%)	
Favored	93.1
Allowed	6.9

Values in parentheses refer to highest-resolution shell.

**Figure 2:**
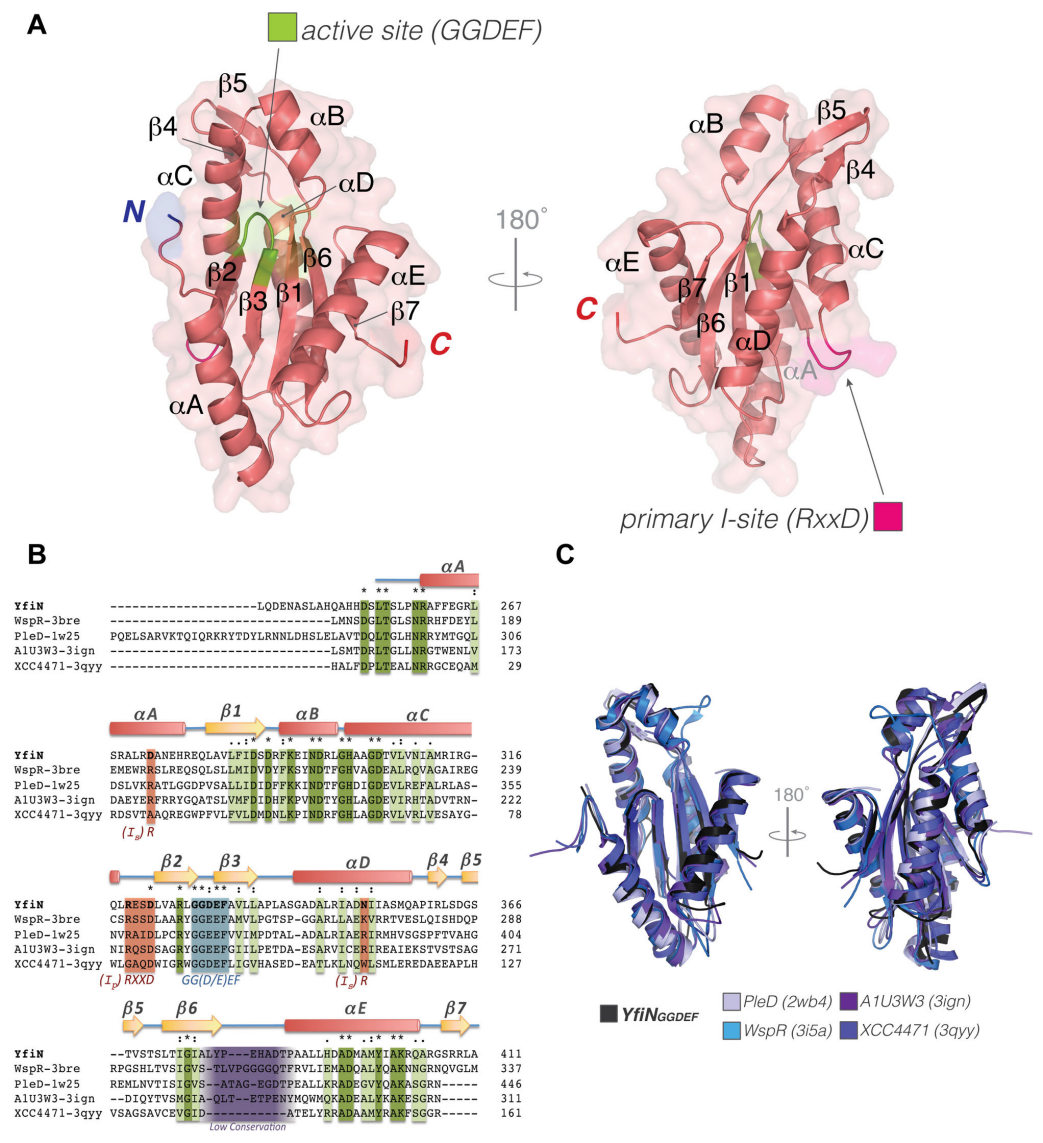
Cristal structure of YfiN_GGDEF_. **A**) Cartoon representation of the YfiN_GGDEF_ structure. The active site and primary inhibitory site (I_p_) signature residues (GGDEF and RxxD) are shown in green and magenta respectively. **B**) Sequence alignment of the GGDEF domain of YfiN with the other DGCs of known structure; PleD from *C. crescentus* [27,28]; WspR from *P. aeruginosa* [29]; A1U3W3 from *M. aquaeolei* [32] and XCC4471 from *X. campestris* [31]. **C**) Structure superposition of YfiN_GGDEF_ with the other DGC. YfiN_GGDEF_ (black); PleD from *C. crescentus* [27,28] (grey - PDB: 2wb4 – rmsd: 1.23 Å); WspR from *P. aeruginosa* [29] (cyan - PDB: 3i5a - rmsd: 1.31 Å); XCC4471 from *X. campestris* [31] (light purple - PDB: 3qyy - rmsd: 1.64 Å) and A1U3W3 from *M. aquaeolei* [32] (dark purple - PDB: 3ign - rmsd: 1.34 Å).

The crystal structure of the catalytic domain of YfiN is composed by a five-stranded β-sheet core (β2-3-1-6-7) flanked by five α-helices (αA to F) (Figure 2). YfiN_GGDEF_ also displays an additional peripheral β-hairpin (β4-5), which is present in all the homologues structures (PleD from *Caulobacter crescentus* [27,28]; WspR from *P. aeruginosa* [29,30]; XCC4471 from *Xanthomonas campestris* [31] and A1U3W3 from *Marinobacter aquaeolei* [32]) with the exception of WspR that displays a long loop in a very different conformation. As expected, the overall scaffold of the structure is similar to the previously solved analogues (Figure 2). However, the cyclase domain of YfiN significantly differs from the other homologues at the level of the allosteric inhibitory site (I-site).

### YfiN displays a degenerated I-site

**Figure 3:**
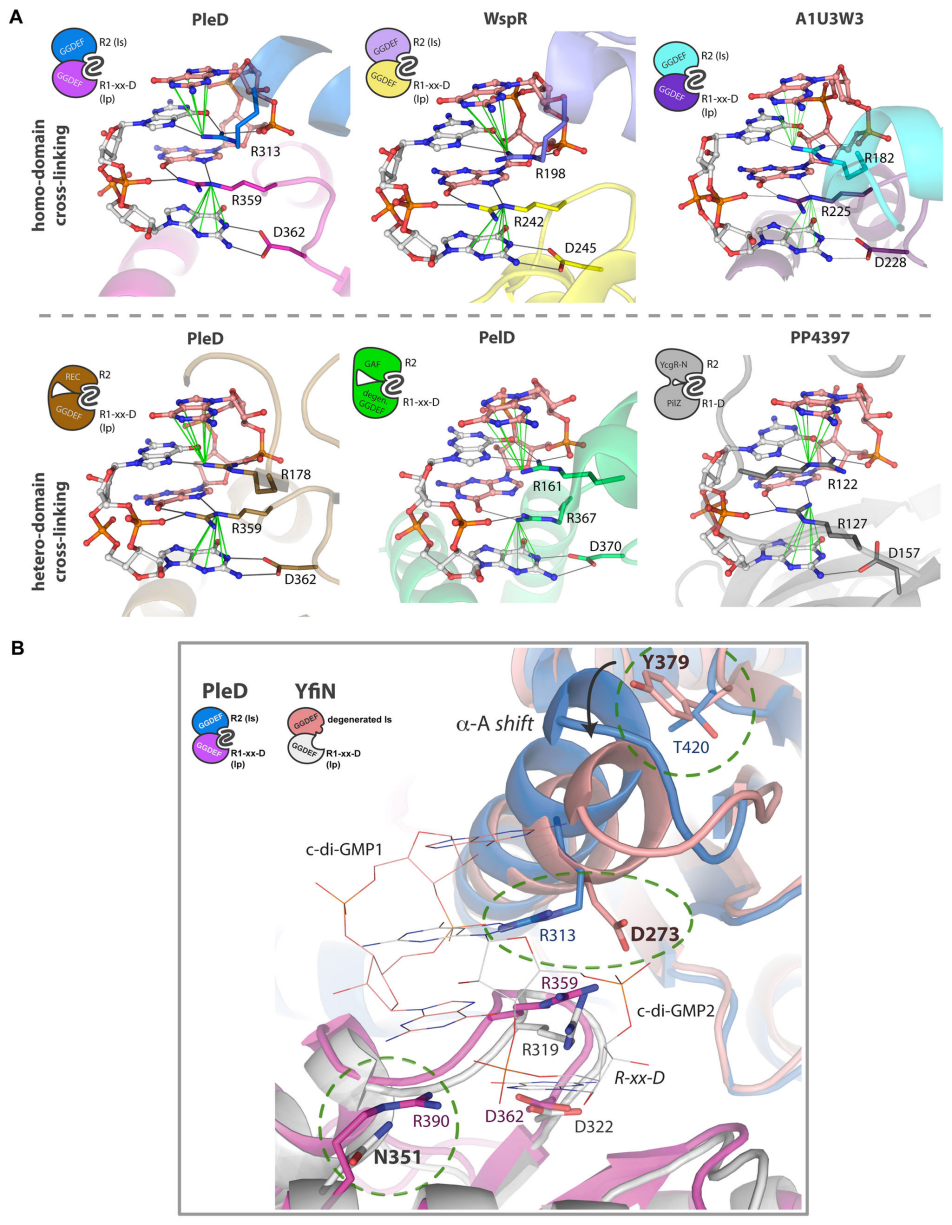
YfiN displays a degenerated I_s_-Site. **A**) Binding mode of dimeric c-di-GMP to the I-site of DGCs or to receptor proteins. The first row shows the homo-domain cross-linking (GGDEF/GGDEF), while the second shows the hetero-domain cross-linking (within the same chain) of inhibited PleD and two c-di-GMP receptors. For all structures different colors are used to illustrate domains belonging to different subunits, the side chains of the two arginines and the aspartic acid (R1; R2 and D) are shown as sticks, while the two bound c-di-GMP molecules as balls and sticks. Grey continuous lines indicate H-bonds, while green continuous lines highlight the π-cation interaction among a charged nitrogen atom of the arginine residues and the guanine delocalised π-system. I_p_ and I_s_ indicate primary and secondary inhibitory sites respectively. Starting from top left, the reported structure are: PleD (PDB: 2v0n [28]); WspR (PDB: 3bre [30]); A1U3W3 (PDB: 3ign [32]); PleD (PDB: 1w25 [27]); PelD (PDB: 4dn0 ) [33]. and PP4397 (PDB: 3kyf [34]). **B**) Comparison of the I-site of YfiN and (PDB: 2v0n [28]). The two subunits of a hypothetical inhibited dimer of YfiN (superposed on the structure of PleD) are shown in white and pink, while the same color code of panel A is used for PleD. C-di-GMP molecules (bound to PleD) are shown as lines. YfiN lacks two of the three arginine residues binding to c-di-GMP through the stair motif interaction (D273 and N351 - bold labels). Moreover, the presence of a bulky side chain (Y379) yields a shift of helix-A, implying a reduced, sub optimal, volume of the I-site.

It is a general feature of DGCs to undergo a negative feedback inhibition caused by the product binding to the so-called I-site. In particular, c-di-GMP binds as a mutually intercalated dimer with sub micro-molar affinity to the DGCs that display a conserved I-site [27,28,30] and the final effect is a cross-link between two domains that hijacks these enzymes to an inactive conformation by spatially separating the two active site. The same binding mode of dimeric c-di-GMP is also observed in receptor proteins as PelD from *P. aeruginosa*, containing a degenerated GGDEF domain [33], or PP4397 from *P. putida*, that displays a PilZ domain [34]. In all cases, enzymes or receptors, when c-di-GMP binds as an intercalated dimer an interlock between two domains is observed. These can be either identical (i.e. GGDEF/GGDEF) or different domains (i.e. GGDEF/REC, GGDEF/GAF, YcgR-N/PilZ) (Figure 3A). Among the many residues that interact with dimeric c-di-GMP in these structures, three are invariantly present: an arginine and an aspartate on one domain and a second arginine on the other domain. In particular, whilst the aspartate is probably involved in ligand recognition and binding, the two arginine residues appear to be crucial for cross-linking to take place (Figure 3A). Essentially, these arginine residues bind c-di-GMP making a π-cation interaction with one guanine while H-bonding a second one. This peculiar binding mode is called *stair-motif* interaction and is recurrent in protein/DNA complexes [35,36]. Each arginine residues interacts with both c-di-GMP molecules. Therefore, since each domain provides one of the two key arginines, dimeric c-di-GMP is able to *glue* two domains through a double stair-motif interaction. In the case of the I-site of DGCs the first arginine is provided by the primary I-site (I_p_) of the GGDEF domain (the conserved RxxD motif), while the second may be recruited from the secondary I-site (I_s_) of another GGDEF domain [28,30,32] or from a different one (i.e. the REC domain of PleD [27] or the receptors PelD [33] and PP4397 [34]). Consequently, it must be clarified that the presence of the RxxD motif in the primary I-site of a DGC domain is a necessary but not sufficient condition for feedback inhibition, since a second arginine, provided by the I_s_ or another domain, is also needed.

The GGDEF domain of YfiN displays a conserved RxxD motif in the I_p_ , while the I_s_ appears degenerated. In particular, the second arginine necessary to form an inactive GGDEF/GGDEF dimer, is substituted with Asp-273 (Figure 3B and 2B). Moreover, another important arginine is missing in YfiN I_s_ . This residue, which in PleD is Arg-390 and buttress (c-di-GMP)_2_ by an additional stair-motif interaction [28], in YfiN is substituted with Asn-351. Finally, the α-helix harboring the I_s_ (α-A) is shifted with respect to the corresponding helix of PleD, WspR and A1U3W3, which all display product feedback inhibition. The shift is due to the hindrance of Tyr-379 side chain (Figure 3B). A similar shift, which hampers potential binding of (c-di-GMP)_2_ to the I-site for sterical reasons, is observed only in the structure of XCC4471 that also displays a degenerated I-site [31].

These evidences suggest that YfiN is not able to undergo canonical product inhibition of DGCs, implying homodimer formation between the two catalytic domains. However, since the RxxD motif is conserved, the enzyme could still bind dimeric c-di-GMP and display product inhibition through an eventual cross-link of the GGDEF and HAMP domain, with the second arginine provided by the latter. To verify this possibility we measured the binding affinity of YfiN_HAMP-GGDEF_ for c-di-GMP.

### YfiN_HAMP-GGDEF_ does not bind c-di-GMP

**Figure 4:**
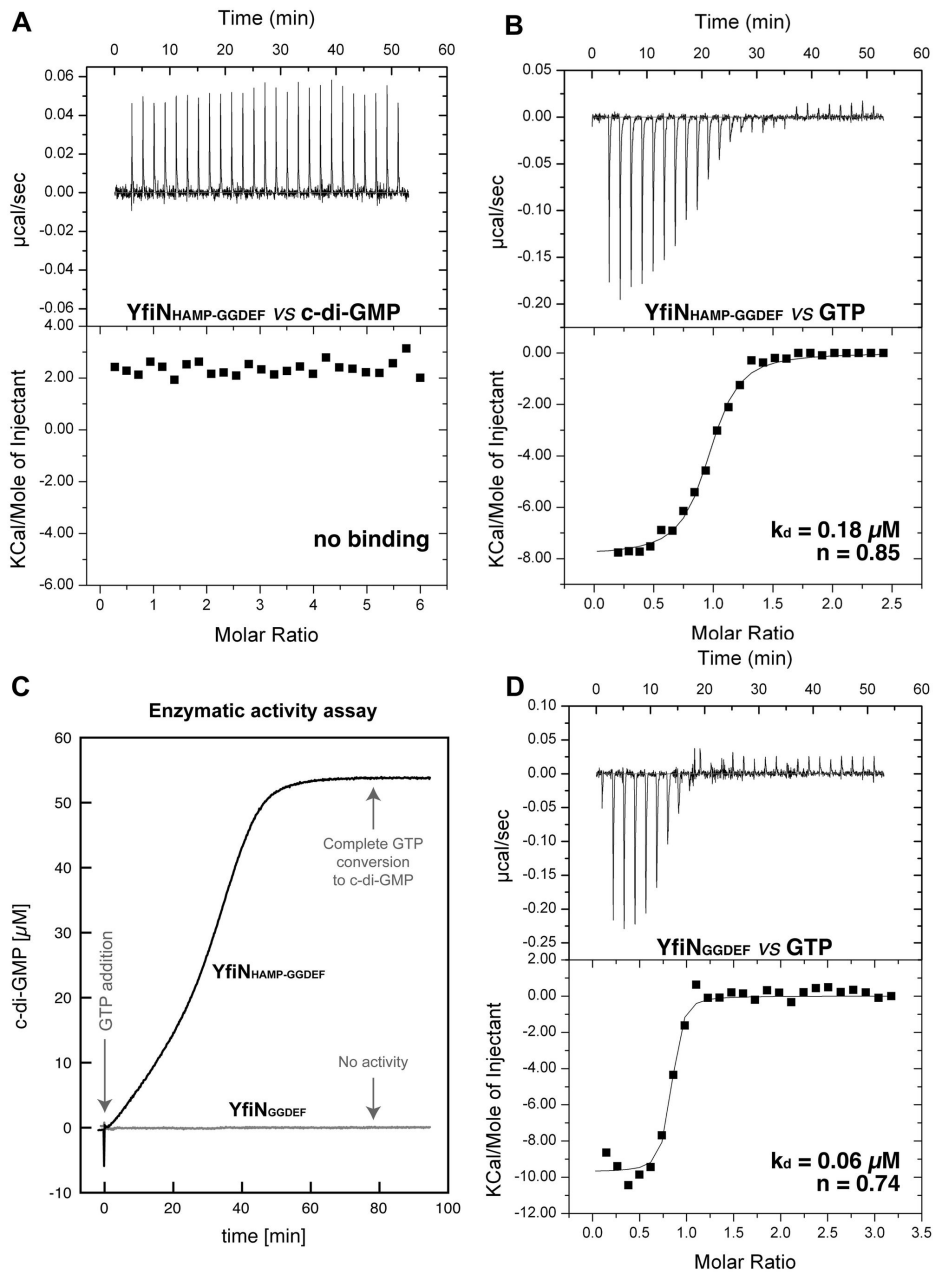
Binding affinity for nucleotides and enzymatic activity of YfiN_HAMP-GGDEF_ and YfiN_GGDEF_. For all ITC experiments upper panels show the Raw ITC data, while lower panels show the integrated peak areas (black square) fitted with the one-binding-site model of ORIGIN provided by MicroCal (continuous lines). Derived thermodynamic parameters are listed in Table 2 A) Microcalorimetric titration of 3 μM YfiN_HAMP-GGDEF_ with c-di-GMP (90 μM in the syringe). No binding was observed either in the presence of CaCl_2_ or in the presence of MgCl_2_/MnCl_2_ (data not shown). No thermodynamic parameters were derived. **B**) Microcalorimetric titrations of 14 μM enzyme solution with GTP (170 μM in the syringe). The thermodynamic profile indicates that the interaction of YfiN_HAMP-GGDEF_ with GTP presents favorable binding enthalpy and entropy, which suggests that hydrogen bonding and hydrophobic interactions are mainly involved in the binding event, rather than conformational changes. **C**) Cyclase activity of 10 µM YfiN_HAMP-GGDEF_ or YfiN_GGDEF_ assayed in real time by circular dichroism spectroscopy after addition of 100 µM GTP. For YfiN_HAMP-GGDEF_ (Black) The final c-di-GMP concentration corresponds to complete conversion of 100 µM GTP, whilst for YfiN_GGDEF_ (grey) no product is detected even if the sample is allowed to react for 24 h (not shown). **D**) Microcalorimetric titrations of 11 μM YfiN_GGDEF_ with GTP (170 μM in the syringe).

Binding of c-di-GMP to YfiN_HAMP-GGDEF_ was directly measured using isothermal titration calorimetry (ITC) and no binding was observed (Figure 4A). Of course an eventual misfolding of the soluble truncated construct could bias this result. To exclude this possibility we also measured the binding affinity of YfiN_HAMP-GGDEF_ for the substrate. Binding of GTP was carried out in the presence of CaCl_2_, which does not allow hydrolysis after substrate binding. YfiN_HAMP-GGDEF_ binds GTP with submicromolar affinity and a stoichiometry close to one (Figure 4B). As expected for specific binding, integration of the titration peaks produced a sigmoidal enthalpy curve for the interaction (the corresponding results are summarized in Table 2). It is worth mentioning that the K_d_ measured in this experiment could not correspond to the K_M_ value, since no catalysis has followed the binding event; moreover, it is not excluded that the affinity of GTP for the active site may be slightly altered by the calcium ion, with respect to the physiological metal (i.e. magnesium or manganese). To verify whether c-di-GMP could in any way hamper or negatively affect substrate binding to YfiN_HAMP-GGDEF_, the GTP binding experiment was also repeated in the presence of an excess of product: no influence of c-di-GMP on the binding affinity of the substrate was observed (Figure S2 and Table 2). Taking these data together we can also exclude an eventual feedback inhibition mechanism involving hetero-domain cross-linking. To further verify whether these results could be affected by the truncation of the N-terminal portion of the enzyme, we measured the enzymatic activity of purified YfiN_HAMP-GGDEF_.

**Table 2:** Thermodynamic parameters derived from Microcalorimetric titrations of YfiN_HAMP-GGDEF_ and YfiN_GGDEF_ with nucleotides.

**Protein**	**Ligand**	**n**	**K_a_ x 10^6^ M^-1^**	**K_d_ µM**	**ΔH kcal/mol**	**−ΤΔS kcal/mol**	**ΔG kcal/mol**
**YfiN_HAMP-GGDEF_**	GTP	0.85 ± 0.1	5.62 ± 1.9	0.18	-8.1 ± 0.3	-1.29	-9.36
**YfiN_HAMP-GGDEF_^*a*^**	GTP	0.73 ± 0.03	6.46 ± 2.7	0.15	-7.1 ± 0.3	-2.24	-9.30
**YfiN_HAMP-GGDEF_**	c-di-GMP	n.d.	n.d.	n.d.	n.d.	n.d.	n.d.
**YfiN_GGDEF_**	GTP	0.74 ± 0.04	18.1 ± 7.5	0.067	-9.9 ± 0.9	-5.31	-10.4

Values are the means of three independent experiments.

*a* This experiment was done after incubation of both GTP and protein samples with 40 µM c-di-GMP.

### YfiN_HAMP-GGDEF_ is active in vitro

The enzymatic activity of YfiN_HAMP-GGDEF_ could be measured using a new method for *in vitro* real-time quantification of c-di-GMP recently developed in our group [23]. We observed complete conversion of GTP to c-di-GMP (Figure 4C). It may be assumed that in order to condensate two GTP molecules, the GGDEF domains must come together at a certain time during catalysis. In this sense, it is important to notice that, although monomeric in solution, the purified YfiN_HAMP-GGDEF_ is still able to catalyze the condensation reaction of two molecules of GTP to c-di-GMP *in vitro*. Therefore, since neither the presence of the substrate nor that of the product changes the oligomeric state of the enzyme (data not shown), the formation of a transient catalytic dimer must be assumed. Indeed, the real time kinetics, as monitored with this new method, displays an interesting sigmoidal behavior (currently under investigation), which may well be related with such a mechanism. To verify the role of the HAMP domain in transient dimer formation, we produced a shorter construct containing only the GGDEF domain (YfiN_GGDEF_; residues 232-435). This construct, which as expected is monomeric (Figure S5), although still able to bind GTP with micro-molar affinity, is completely inactive (Figure 4C and 4D), indicating that the HAMP domain is crucial for transient dimerization and catalysis to occur. On the other hand, the activity of YfiN_HAMP-GGDEF_ confirms that YfiN does not undergo product feedback inhibition, at least *in vitro* and in the micromolar range that we explored (up to 50 µM c-di-GMP). Likewise, Wood and co-workers have shown that *in vitro* feedback inhibition for full-length YfiN is observed only at c-di-GMP concentration higher than 200 μM [18].

Thus, the YfiBNR signaling system appears to be an ON/OFF switch, with the output of the module (i.e. c-di-GMP production) responding only to external stress signals and not to endogenous c-di-GMP levels. It as been shown that the domain architecture of YfiN represents a widespread module to connect periplasmic stimuli to a cytosolic response or *vice versa* [14,37–39]. It is, therefore, compelling to clarify the molecular detail of this allosteric inside-out signaling system.

### Homology modeling of full-length YfiN

**Figure 5:**
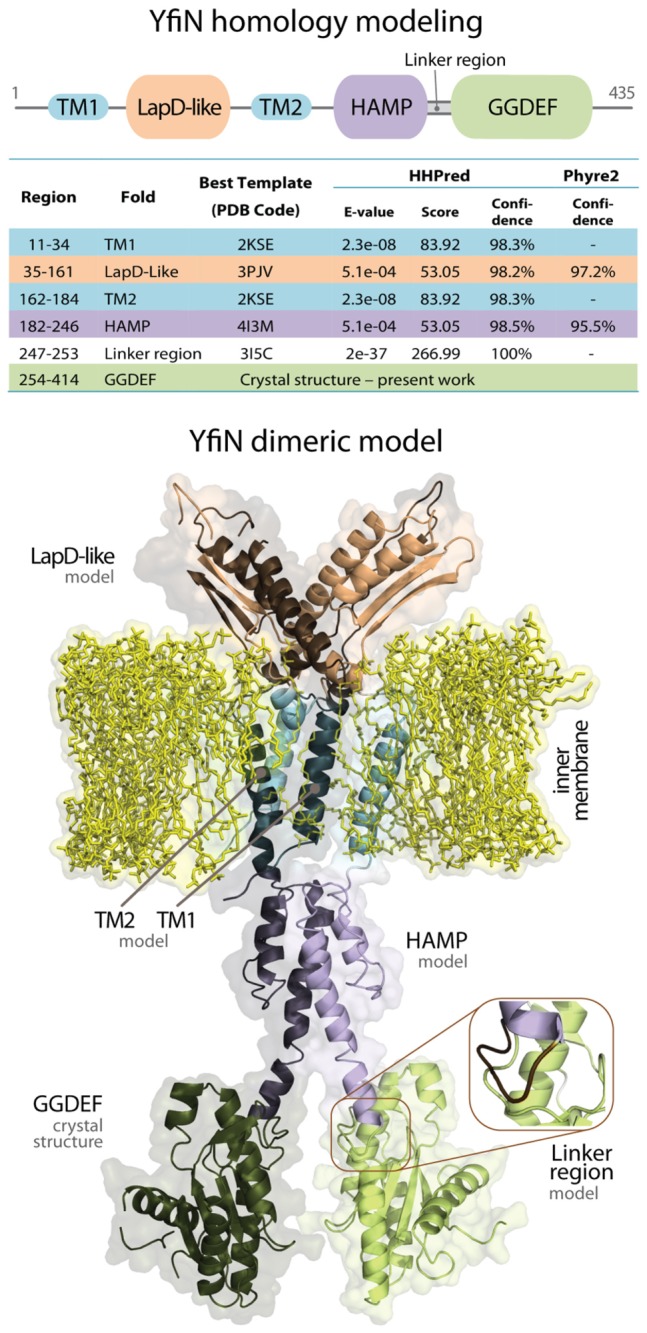
Dimeric model of YfiN. Predicted domain organization of YfiN along with the most significant structural templates found, according to two different fold prediction servers (i.e., Phyre2 [25] and HHPRED [26]) used for homology modeling. The final model including the crystal structure of the catalytic domain is also shown.

To gain insights into the mechanism of allosteric regulation of YfiN and how modifications affecting the periplasmic domain are transmitted into the cytoplasm, homology modeling of the full-length dimeric protein was attempted. Figure 5 shows the predicted domain organization of YfiN along with the most significant structural templates found, according to two different fold prediction servers (i.e., Phyre2 [25] and HHPRED [26]), and the dimeric model of YfiN. The N-terminal region of YfiN has been previously predicted to fold as a PAS domain, and consequently modeled [20] using as structural template the Sensor Kinase CitA binding domain (PDB Code: 1p0z [40]). However, the recent finding that the N-terminal domain of the HAMP-GGDEF-EAL protein LapD from *P. fluorescens* adopts a novel fold, consisting of a V-shaped, domain-swapped dimer (PDB Code: 3pjv [24]) that shows only weak structural similarity to the PAS fold (RMSD ~2.5 Å), prompted us to investigate further this issue by resubmitting the N-terminal region of YfiN to HHPRED and another fold prediction method, Phyre2 [25]. Consistent with our premise, residues 35-161 of YfiN are predicted to fold as a swapped LapD-like domain with a score and significance (HHPRED: E-value = 5.1 e-04, score = 53.05, confidence = 98.2%; Phyre2: confidence = 97.2%) higher compared to the Sensor kinase CitA (HHPRED: E-value = 1.3, score = 33.59, confidence = 91.2%). Each arm of this fold consists of two α-helices and two β-strands contributed by one of the two protomers, complemented by two β-strands flanked by helical segments from the other [24]. As in LapD, the N- and C-terminal helices of the LapD-like domains presumably connect directly to the transmembrane helices (TM2) and the HAMP domains. To model the later domain (residues 182-246) we used as structural template the HAMP domain of the aerotaxis transducer AER2 (PDB Code: 4I3M [39]), while transmembrane helices and neighboring positively charged loop regions (residues 11-34; 162-184) were modeled based on Sensor protein QSEC (PDB Code: 2KSE [41]), for all alignments see Figure S3. Finally, the model was connected to the crystal structure of the C-terminal GGDEF domain by modeling the linker region (residues 247-253) on the basis of the template diguanylate cyclase response regulator WspR (PDB Code: 3I5C [29]). 

**Figure 6:**
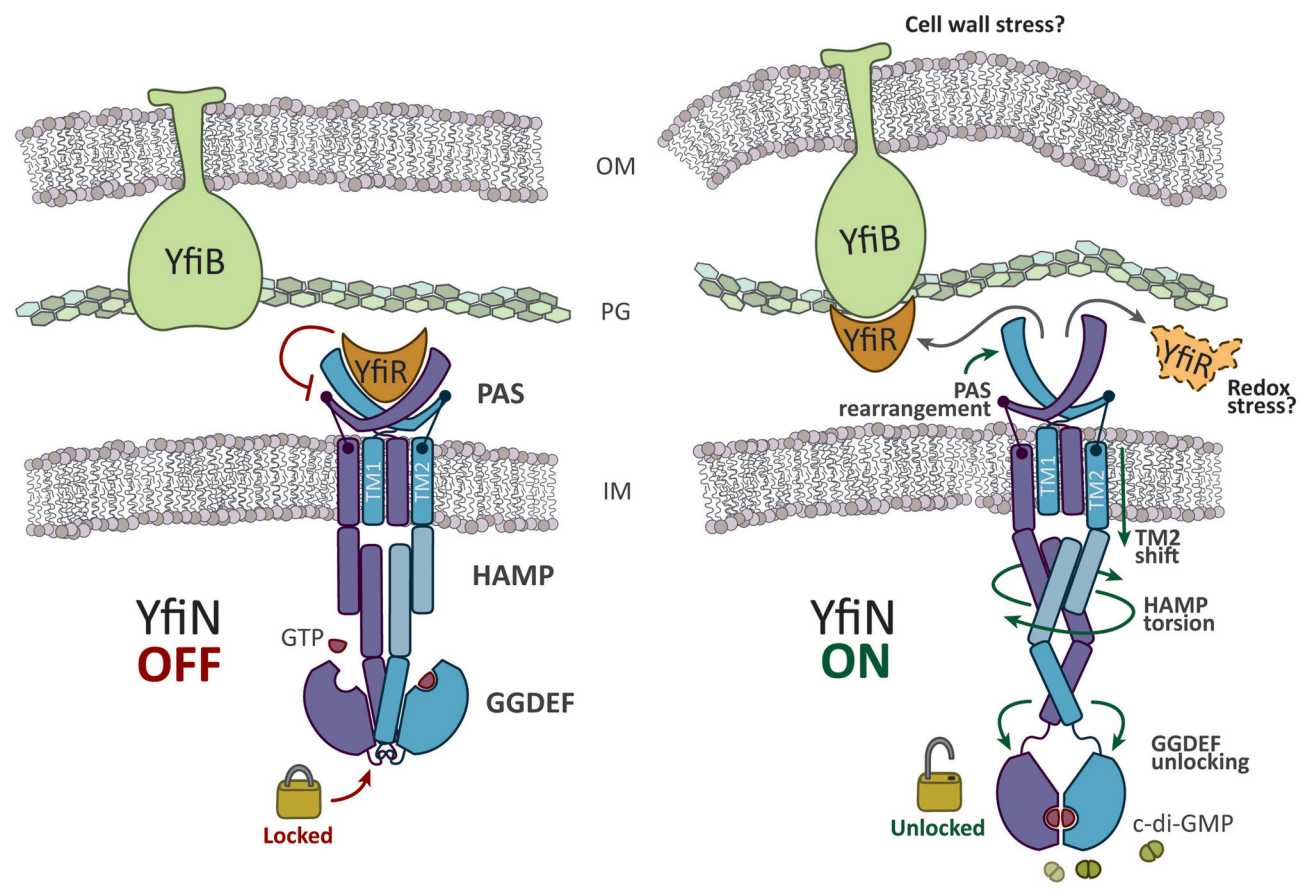
Scheme of allosteric regulation of YfiN. Schematic representation of the putative allosteric regulation of YfiN based on homology modeling pointing to a LapD-like allosteric communication between the periplasmic and the cytosolic portions of the enzyme that is mediated by a conformational change of the HAMP domain.

Following the results of the homology modeling it is likely that the allosteric switch of YfiN resembles that suggested for the LapD receptor [24]. In particular, as illustrated in Figure 6, YfiR would bind in the central gorge of the V-shaped PAS domain of YfiN’s dimer. The release of the complex should produce a conformational change of the two arms of the PAS domains resulting in a shift of the TM2 helices, which are pushed towards the cytosolic side of the inner membrane. This movement of the TM2 should then be transmitted through a torsion of the HAMP domains helices to the terminal of this allosteric chain that is the conserved linker region connecting the last α-helix of the HAMP (*stalk helix*) to the GGDEF domain. The final effect is the unlocking of the C-terminal domains, which are now able to adopt a catalytically competent dimeric conformation (Figure 6). 

### Normal modes and sequence conservation analyses are in agreement with the allosteric regulation model of YfiN

To support this hypothetical mechanism, we analyzed the conformational changes and hinge regions of YfiN, underpinning its allosteric regulation. To this end, we applied coarse-grained, residue-level elastic network models (namely, the Gaussian Network Model [GNM] and its extension Anisotropic Network Model [ANM] [42,43]) to the full dimeric model of YfiN. Movie S1 provides a convenient visualization of the obtained results. The predicted LapD-like domain of YfiN undergoes a very large conformational bending, varying the angle between the arms of the V-shaped fold, most likely as a consequence of YfiR binding. Such a bending triggers, through the movement of the TM2 helices and the first predicted hinge region (residues 153-154), a torsional rotation of the downstream HAMP domain, which could form therefore the structural basis for modulating the interaction between the C-terminal GGDEF domains, possibly through an unlocking of the second predicted hinge, the linker region (residues 247-253).

**Figure 7:**
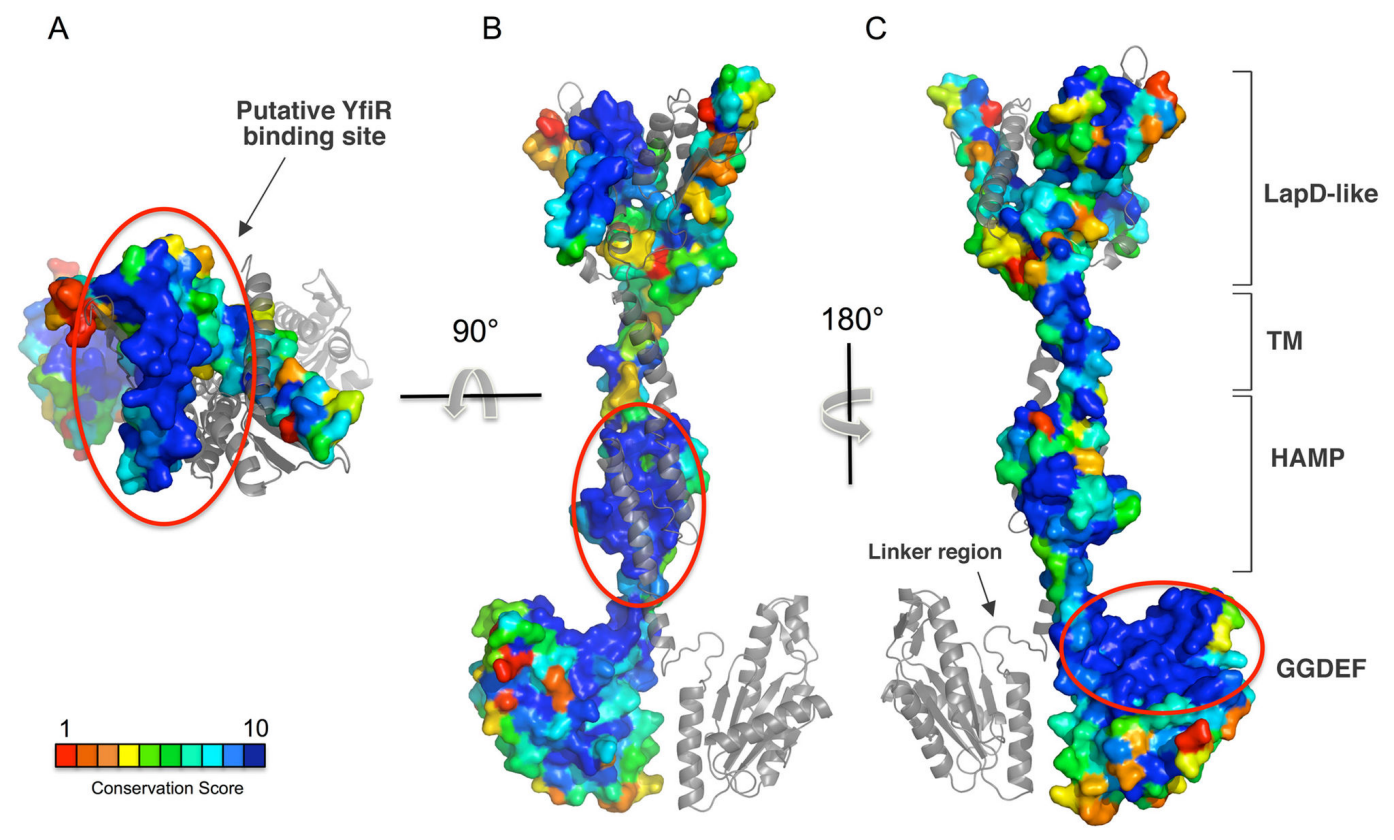
Mapping sequence conservation on YfiN model. Location of strictly conserved regions (grading from cyan to blue) mapped on the model of YfiN. **A**) The central V-shaped gorge of the periplasmic domain is fully conserved. Since this region is solvent exposed a similar conservation degree suggests that this is the putative binding site of YfiR. **B**) The core of the four-helices bundle of the HAMP domain is conserved, as expected. **C**) The most conserved region of the GGDEF domain comprises the region of the active site (highlighted in red) and the linker region, the small loop connecting the catalytic and the HAMP domains. The conformation of the linker region, as modeled on the structure of WspR [29]), would not allow the two GGDEF domain to assume catalytically competent conformation (i.e. with the two active sites facing each other). Therefore a severe rearrangement of the linker region (unlocking) must be assumed in order for catalysis to occur.

As an additional indirect support to this hypothetical mechanism, we mapped the sequence conservation of YfiN and the position of known activating/inactivating mutations [20] on the full length model of YfiN, to confirm the potentially important regions for activity and/or allosteric regulation (Figure 7). Therefore, a multiple sequence alignment of 53 non-redundant orthologous of YfiN sequences was constructed from other Pseudomonas strains and from more distantly related sequences from other bacteria (Figure S4).

Strikingly, the accessible central gorge of the LapD-like periplasmic domain, presumably involved into the interaction of the periplasmic domain with YfiR, is characterized by a well-conserved helix spanning residues 44-72 (aLrxYaxxNlxLiaRsxxYTxEaavvFxD; Figure 7A). This region not only is highly exposed but also includes 90% of the identified mutations in the periplasmic domain of YfiN that produce YfiR-independent alleles (residues 51, 58-59, 62, 66-68, 70) [20].

The folding of the dimeric HAMP domains as a four-helices bundle is also supported by the strict conservation of the core of the helix-loop-helix motif putatively involved in dimerization with the other monomer (residues 216-235: ELxxlxxDFNxLxdElexWq; (Figure 7B). Interestingly, since both YfiN_HAMP-GGDEF_ and YfiN_GGDEF_ constructs are monomeric in *in vitro* and bind GTP with similar affinity, but only the first is able to further condensate it to c-di-GMP, we must assume that, for YfiN_HAMP-GGDEF_, catalysis proceeds through a HAMP-mediated transient dimerization. Therefore, we can speculate that the periplasmic domain of YfiN may not only play a regulatory role, but would also be essential to maintain the enzyme in a dimeric state, allowing the HAMP domains to form a stable four-helices bundle, thus keeping the two GGDEF domains in close proximity.

The linker region between the C-terminal GGDEF domain and the stalk helix of the HAMP domain, that we suggest to be crucial in the allosteric regulation, is also highly conserved (residues 249-260: AxHDxLTgLxNR) (Figure 7C). The importance of this region is confirmed by the deletion mutant ∆255-257, which is inactive and is dominant over the activating substitution G173D [20]. We have modeled this loop on the basis of the inhibited structure of WspR (PDB Code: 3I5C [29]) but, based on the location of the GTP binding site, this conformation would be incompatible with a catalytic encountering of the two GGDEF domains. Therefore, a severe rearrangement of this region, as a consequence of the HAMP domains torsion, must be assumed for catalysis to take place. Thereby, the role of the linker region would be to allosterically allow or deny the encountering of the two GGDEF domains depending on the HAMP conformation. Moreover, since this linker loop is located near the substrate binding site, it is not excluded that GTP binding may also play a role in the conformational change of this region of the enzyme.

Finally, the C-terminal GGDEF domain is also characterized by a large evolutionarily conserved surface region, which comprise the active site GGDEF motif (residues 319-338: RexDxVaRlGGDEFavllxp), and the adjacent helix-turn-helix region (residues 290-310: DxDxFKxxNDxxGHaxGDxVL;) (Figure 7C). These are presumably involved in GTP binding and monomer-monomer contacts upon formation of the catalytically competent GGDEF dimer.

## Conclusions

We have shown that YfiN displays a degenerated secondary I-site and that the conserved primary I-site (RxxD) has no counterpart supplied by the HAMP domain, since YfiN_HAMP-GGDEF_ is not able to bind c-di-GMP. On the other hand, YfiN_HAMP-GGDEF_ binds GTP with sub-micromolar affinity, and is able to condensate it into c-di-GMP. These data point to the conclusion that YfiN does not undergo product feedback inhibition as other DGCs and, therefore, functions as ON/OFF cyclase responding solely to periplasmic signals.

It is becoming clear that the regulation of different DGCs depends firmly on the architecture of the accessory domains of each enzyme. Therefore, targeting the allosteric modules (e.g. the regulatory domains) together with of the catalytic domain could become a winning strategy to fight biofilm-mediated infections. This is especially true in the case of the YfiBNR system, which functions as an entry point for different environmental signals during *Pseudomonas* adaptation. Of course, availability of structural data represents the bottleneck for an efficient drug design approach: understanding the structural details of the allosteric control of DGC activity is highly desirable yet challenging. By assuming a LapD-like fold for YfiN periplasmic portion, we could speculate that its allosteric regulation is similar to the P. *fluorescence* receptor [24]. Normal modes and sequence conservation analyses, as well as mapping of the activating/inactivating mutations on the homology model are in agreement with a LapD-like activating mechanism, solely depending on the interaction between YfiR and YfiN in the periplasmic space. Based on our biochemical data on the truncated constructs, indicating that the presence of the HAMP domain is essential to induce the transient dimerization of the monomeric YfiN_HAMP-GGDEF_, we suggest that the periplasmic domain of the full-length protein, by assuming a LapD-like fold that is based on domain-swapping, could function as the driving force for dimerization. A key role in the conformational transition appears to be played by the region connecting the HAMP to the GGDEF domain. We propose that this linker loop may act as a hinge whose locking/unlocking equilibrium, driven by the conformation of the HAMP domain helices, controls the catalysis by keeping the two GGDEF domains separated or allowing their facing (Figure 6). Catalysis through transient encountering of the GGDEF domains could be a general feature of DGCs, which have evolved different regulatory modules that inhibit catalysis always by spatially separating the two GGDEF domains [27,29]. On the other hand, the GGDEF domains are dynamically exploring their allowed conformational space looking for each other like lovers do, waiting for activation and substrate to come and let them finally meet.

## Materials and Methods

### Protein cloning, expression and purification

Both the *YfiN*_*HAMP-GGDEF*_ and *YfiN*_*GGDEF*_ fragments were amplified from a pET24b plasmid harboring a synthetic *YfiN*_*fl*_ gene (Geneart). The purified PCR products, verified by sequencing, were ligated (NdeI, XhoI) in frame with a C-terminal His-tag into a pET24b vector (Novagen) and transformed into BL21-(DE3) *E. coli* strain for expression.

Both construct were expressed as described in [14]. Briefly: cells from a single colony were used to inoculate 5 mL of Luria-Bertani (LB) medium containing 30 μg/mL of kanamycin and grown at 37° C. After 10 h cells were diluted into 300 mL of LB and grown at 37° C over night before final dilution in 3x1 L of LB. Cells were grown for 2.5 h at 37° C before induction with 100 µM isopropyl β-D-1-thiogalactopyranoside (IPTG). After 2.5 h at 30° C cells were harvested by centrifugation and stored at -20 °C.

Cells were lysed by sonication and proteins were purified using an Ni-HiTrapTM Chelating HP column (GE Healthcare) equilibrated with 10 mM Tris–HCl, pH 8.0, 250 mM NaCl, 10% glycerol; the proteins were eluted with 100 mM imidazole, in the same buffer. Finally, the purified proteins were loaded on an FPLC column (Superdex 75 10/300, GE Healthcare), and eluted with 10 mM Tris–HCl pH 8.0, 100 mM NaCl, 2% glycerol. Size exclusion chromatography (SEC) analysis for the shorter construct (YfiN_GGDEF_; Mw = 23.5 kDa) indicated an apparent molecular mass of 28 kDa consistent with a monomeric state, while for the YfiN_HAMP-GGDEF_ resulted in an ambiguous apparent molecular mass of 41 kDa, in between a monomeric (28 kDa) and a dimeric (56 kDa) form in solution. Therefore, further investigation of the aggregation state of was conducted on YfiN_HAMP-GGDEF_ by analytical ultracentrifugation (AUC) (Figure S5).

### Analytical Ultracentrifugation

Size distribution of YfiN_HAMP-GGDEF_ in solution was assessed in sedimentation velocity experiments carried out on a Beckman XLI analytical ultracentrifuge using absorbance optics. The experiments were conducted at 35,000 rpm and 20 °C at a protein concentration of 2 mg/mL in 250 mM NaCl, 10 mM Tris-HCl pH 8.0, 10% glycerol. Radial absorbance scans were obtained at 280 nm at a spacing of 0.003 cm with three averages in a continuous scan mode. Sedimentation coefficients were calculated using the software Sedfit [44] and were reduced to water and 20 °C (*s*_20,w_) using standard procedures. Sednterp software (http://sednterp.unh.edu/) was used to calculate the buffer density and viscosity. The sedimentation coefficient (S) of YfiN_HAMP-GGDEF_ was 2.3 for 98% of the protein, consistent with a molecular mass of 21 kDa, pointing to a monomeric state of YfiN_HAMP-GGDEF_ in solution.

### Crystallization - data collection and refinement

Crystallization condition for YfiN_HAMP-GGDEF_ were screened using a crystallization robot (Phoenix, Art Robbins), by mixing 300 nL of 3.7 mg/mL protein solution in 0.1 M NaCl, 10 mM Tris pH 8 and 2 % glycerol with equal volumes of screen solution. No positive hit was observed during the first three month. After seven month one single hexagonal crystal was observed in the droplet corresponding to solution n.17 of Crystal-Screen2 (Hampton) containing 0.1 M Sodium Citrate dehydrate pH 5.6 and 35% v/v tert-butanol. The crystal was flash frozen in liquid nitrogen, without any cryoprotectant, and diffracted to 2.77 Å resolution (ESRF, ID 14.1). Data were processed with XDS [45]. The crystal belonged to the P6_5_22 space group with the following unit cell constants: a=b=70.87 Å; c=107.62 Å.

The Matthews coefficient for YfiN_HAMP-GGDEF_ was 1.38 Å^3^Da^-1^ with a solvent fraction of 0.11, pointing to the assumption that only the GGDEF domain (YfiN_GGDEF_) was present in the crystal lattice (Matthews coefficient for YfiN_GGDEF_ was 1.93 Å^3^Da^-1^ with a solvent fraction of 0.36). Phases were obtained by molecular replacement using the GGDEF domain of PleD (PDB ID: 2wb4) as template with Molrep [46]. Cycles of model building and refinement were routinely carried out with Coot [47] and Refmac5.6 [48], model geometry was assessed by ProCheck [49] and MolProbity [50]. Final statistics for data collection and model building are reported in Table 1. Coordinates have been deposited in the Protein Data Bank (PDB: 4iob).

### ITC analysis

ITC experiments were carried out using an iTC200 microcalorimeter (MicroCal), by titrating YfiN_HAMP-GGDEF_ protein sample with either GTP or c-di-GMP, and YfiN_GGDEF_ with GTP. Nucleotide stock solutions were prepared in water and diluted into ITC buffer (final concentrations: 10 mM Tris pH 8, 250 mM NaCl, 1,7 % glycerol, 5 mM CaCl_2_). Protein solution was diluted into the same buffer lacking glycerol. Titration with c-di-GMP were carried out by injecting 1.5 μL aliquots of 90 µM c-di-GMP to a 3 μM protein solution at 25° C; titration with GTP was carried out by injecting 1.5 μL aliquots of 170 µM GTP to 14 μM protein solution at 25° C. The same experiment has been repeated by incubating both GTP and protein samples with 40 µM c-di-GMP. Injection of nucleotides into buffer was also performed as control, under the same experimental conditions. If indicated, data were fitted as described in [51]. All measurements were done in duplicate and the derived thermodynamic parameters are reported in Table 2.

### Real-time enzymatic essay

YfiN activity was measured by circular dichroism (CD) spectroscopy as described in [23]. In brief: c-di-GMP concentration in solution can be deduced by the specific CD signal of the intercalated c-di-GMP dimer at 282 nm. This signal is enhanced in the presence of manganese, which forms a stable complex with c-di-GMP cis-dimer that is linearly dependent on c-di-GMP concentration. The condensation reaction was started by adding 100 µM GTP (Sigma) to a 10 µM solution of YfiN_HAMP-GGDEF_ or YfiN_GGDEF_ in 150 mM NaCl, 20 mM Tris/HCl pH 7.5, 10 mM MgCl_2_, 2.5 mM MnCl_2_ and 1% glycerol. C-di-GMP formation was monitored following the CD signal at 282 nm, using a 1 cm quartz cuvette (Hellma) on a JASCO J-710 spectropolarimeter at 20° C.

### Homology modeling and in silico analysis

The YfiN protein sequence from *Pseudomonas aeruginosa* was retrieved from the Uniprot database (http://www.uniprot.org; accession number: Q9I4L5). UniRef50 was used to find sequences closely related to YfiN from the Uniprot database. 123 orthologous sequences displaying a minimum percentage of sequence identity of 50% were obtained. Each sequence was then submitted to PSI-Blast (www.ncbi.nlm.nih.gov/blast; number of iterations, 3; E-Value cutoff, 0.0001 [52]), to retrieve orthologous sequences from the NR_PROT_DB database. Sequence fragments, redundancy (>95%) and too distant sequences (<35%) were then removed from the dataset. At the end of this procedure, 53 sequences were retrieved (Figure S4). The conservation of residues and motifs within the YfiN sequences was assessed through a multiple sequence alignment, using the ClustalW tool [53] at EBI (http://www.ebi.ac.uk/clustalw).

Secondary structure predictions were performed using several tools available, including DSC [54] and PHD [55], accessed through NPSA at PBIL (http://npsa-pbil.ibcp.fr/), and Psi-Pred (http://bioinf.cs.ucl.ac.uk/psipred [56]). A consensus of the predicted secondary structures was then derived for further analysis.

A fold prediction-based approach was utilized to gain some structural insights into the domain organization of YfiN and related proteins. Although three-dimensional modeling performed using such techniques is seldom accurate at the atomic level, the recognition of a correct fold, which takes advantage of the knowledge available in structural databases, is often successful. The programs Phyre2 [25] and HHPRED [26] were used to detect domain organization and to find a suitable template fold for YfiN. All the programs options were kept at default.

A three-dimensional model of YfiN (residues 11-253) was constructed using the MODELLER-8 package [57], using as structural templates the following crystal structures: the N-terminal domain of the HAMP/GGDEF/EAL protein LapD from *P. fluorescens* (residues 35-161; PDB Code: 3pjv [24]); the HAMP domain of Aerotaxis transducer AER2 (residues 182-246; PDB Code: 4i3m [39]); Sensor protein QSEC (residues 11-34; 162-184; PDB Code: 2kse [41]); diguanylate cyclase response regulator WspR (residues 247-253; PDB Code: 3i5c [29]).

A full YfiN dimeric model was built starting from the crystal structure of the cyclase domain (GGDEF – present work) and performing a backward multi-step homology modeling approach, in which each new predicted domain has been linked to the previously obtained model by following the orientation of its structural template. The structural templates were oriented as follows: 1) GGDEF domain of YfiN (residues 254-414) was initially superposed to the GGDEF domain of WspR from *Pseudomonas aeruginosa* (PDB Code: 3i5c) to predict the structure and orientation of the linker region (residues 247-253 of YfiN, corresponding to residues 170-176 of 3i5c); 2) the helical stalk motif of 3i5c (residues 157-170) was then superposed to the C-terminal helix of the HAMP domain of the aerotaxis transducer Aer2 (residues 138-156), to predict the structure and orientation of the HAMP domain of Yfin (residues 182-146); 3) the orientation of the TM helices of Sensor protein qseC (PDB Code: 2KSE) with respect to the hydrocarbon core of the lipid bilayer was derived from the OPM server [58]; the N-terminal domain of LapD (PDB Code: 3pjv) was roughly oriented perpendicular to the lipid bilayer, following the relative position of the inner cell membrane and connection to the flanking TM helices as indicated by [24]. Ten different models were built and evaluated using Prosa2003 [59]: the model displaying the lowest energy profile (Z-Score= -4.86) was taken as the representative one. The initial alignment, obtained from threading methods, was then subjected to minor changes in the attempt to increase low score-regions.

Normal mode analysis and hinge regions predictions were carried out by using the “HingeProt” server, using as cutoff distances for GNM and ANM the default values 10 Å and 18 Å, respectively [60].

Evolutionary sequence conservation was mapped onto the accessible surface of the best model by means of CAMPO [61], using the previously obtained alignment.

## Supporting Information

Figure S1 **Residues visible in the crystal structure of YfiN_GGDEF_.** The predicted HAMP and GGDEF domains are underlined in purple and orange respectively. The residues that are visible in the electron density are highlighted in green (254-414). The linker region between the HAMP and the GGDEF domains, where proteolysis conceivably occurred, is coloured in blue.
(TIFF)

Figure S2 **Binding of GTP to YfiN_HAMP-GGDEF_ in the presence of c-di-GMP.** Representative microcalorimetric titration of 14 μM enzyme with GTP (170 μM in the syringe) in the presence of 40 µM c-di-GMP in both solutions. *Upper* *panel*: Raw ITC data. *Lower* *panel*: Integrated peak areas (black square). Fit with the one-binding-site model of ORIGIN provided by MicroCal (continuous lines) is also depicted.
(TIFF)

Figure S3 **Best templates for homology modelling of full length YfiN.** Sequence alignments based on secondary structure prediction of the different domains of YfiN with the most significant structural templates according to two different fold prediction servers (Phyre2 and HHPRED).
(TIF)

Figure S4 **Sequence conservation.** Multiple sequence alignment of 53 non-redundant orthologous of YfiN sequences, from other *Pseudomonas* strains and from more distantly related sequences from other bacteria.
(PDF)

Figure S5 **Determination of the aggregation state of YfiN_HAMP-GGDEF_ and YfiN_HAMP-GGDEF_ in solution.** **A**) Size exclusion chromatography (SEC) of YfiN_HAMP-GGDEF_ (*green*) and YfiN_GGDEF_ (*blue*) after the affinity chromatography purification step. The proteins elutes with an apparent molecular mass of 41 kDa and 28 kDa respectively. **B**) Calibration curve obtained using the following standards: BSA 66 kDa; Carbonic Anhydrase 29 kDa; Myoglobin 18 kDa; Ribonuclease A 13.7 kDa and Aprotinin 6.5 kDa. **C**) Sedimentation velocity experiment to determine the size distribution of YfiN_HAMP-GGDEF_ in solution. The sedimentation coefficient (S) was 2.3 for 98% of the protein, consistent with a molecular mass of 21 kDa, and indicating a monomeric state of YfiN_HAMP-GGDEF_ in solution. **D**) The YfiN_HAMP-GGDEF_ , the results of the SEC analysis indicates that the two domains of the protein are mobile, thus displaying a large hydrodynamic volume. On the contrary, YfiN_GGDEF_ displays an apparent molecular mass consistent with a monomer, as illustrated in the scheme.
(TIF)

Movie S1 **Normal Modes Analysis on YfiN model.** The animation illustrates the rigid parts of YfiN the hinges connecting them, together with the direction of the fluctuation of each residue in the slowest two modes as predicted by the server *HingeProt* [60]. Two orthogonal points of view of the predicted protein motion are shown on the left and on the right respectively.
(MOV)
