# Supplementary figures and images for: Investigating the Allosteric Regulation of YfiN from Pseudomonas aeruginosa: Clues from the Structure of the Catalytic Domain
Source: PLoS One. 2013 Nov 22;8(11):e81324. doi: 10.1371/journal.pone.0081324 (PMC3838380; doi:10.1371/journal.pone.0081324)

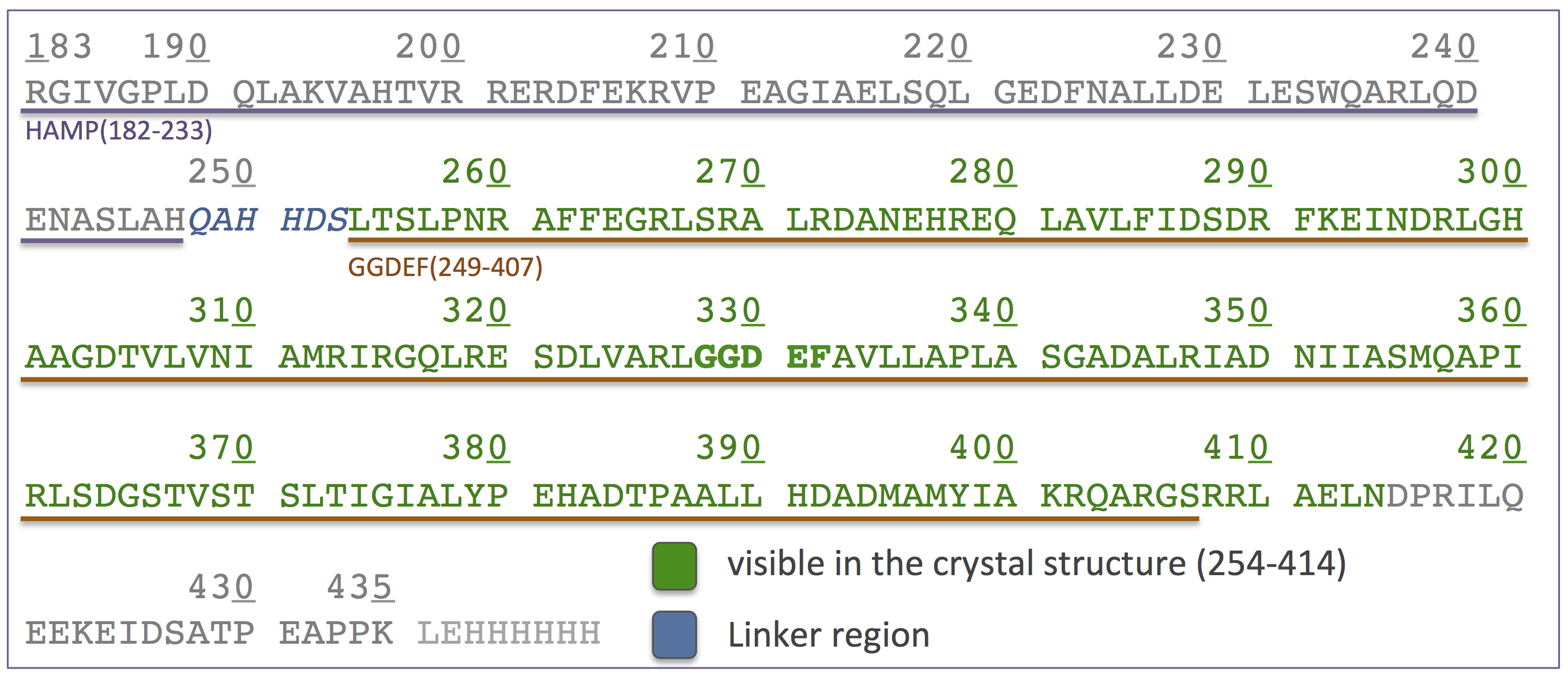

Supplement: Figure S1 — Residues visible in the crystal structure of YfiNGGDEF. The predicted HAMP and GGDEF domains are underlined in purple and orange respectively. The residues that are visible in the electron density are highlighted in green (254-414). The linker region between the HAMP and the GGDEF domains, where proteolysis conceivably occurred, is coloured in blue. (TIFF) [file pone.0081324.s001.tiff]

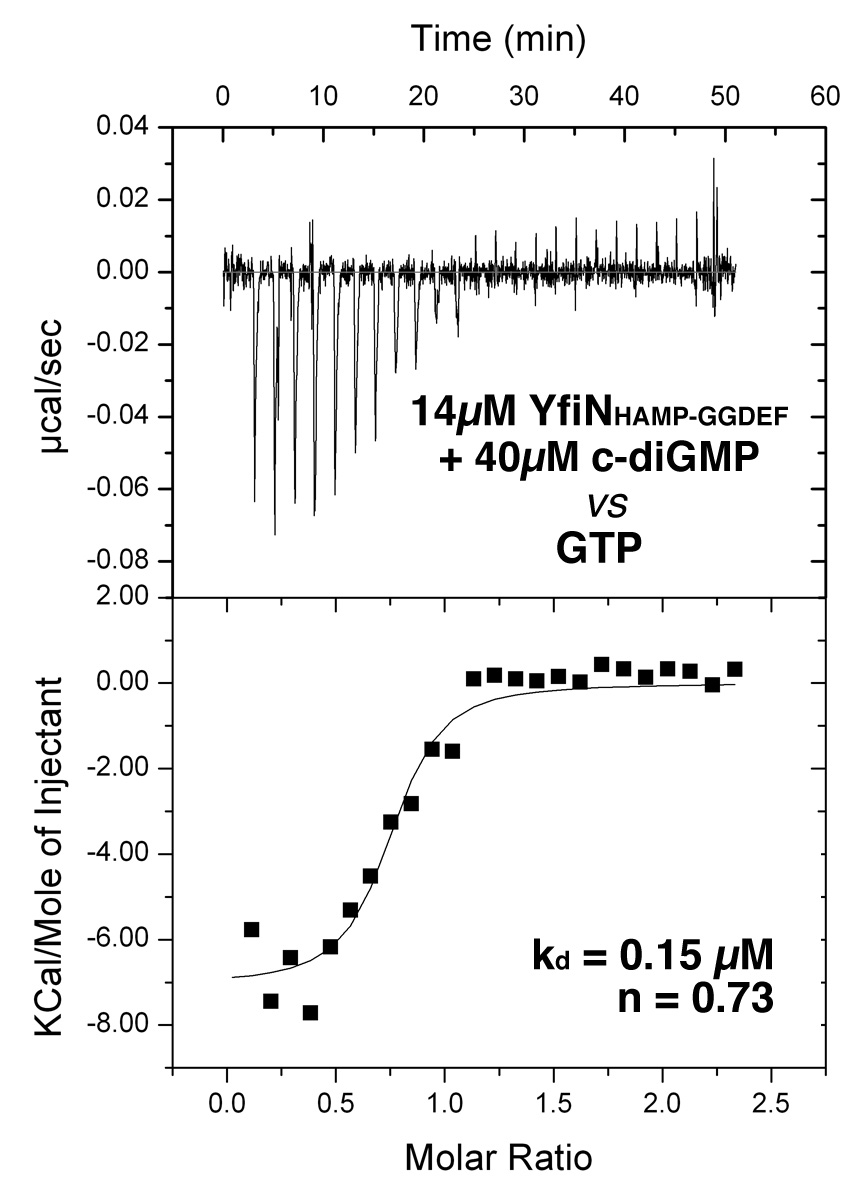

Supplement: Figure S2 — Binding of GTP to YfiNHAMP-GGDEF in the presence of c-di-GMP. Representative microcalorimetric titration of 14 μM enzyme with GTP (170 μM in the syringe) in the presence of 40 µM c-di-GMP in both solutions. Upper panel: Raw ITC data. Lower panel: Integrated peak areas (black square). Fit with the one-binding-site model of ORIGIN provided by MicroCal (continuous lines) is also depicted. (TIFF) [file pone.0081324.s002.tiff]

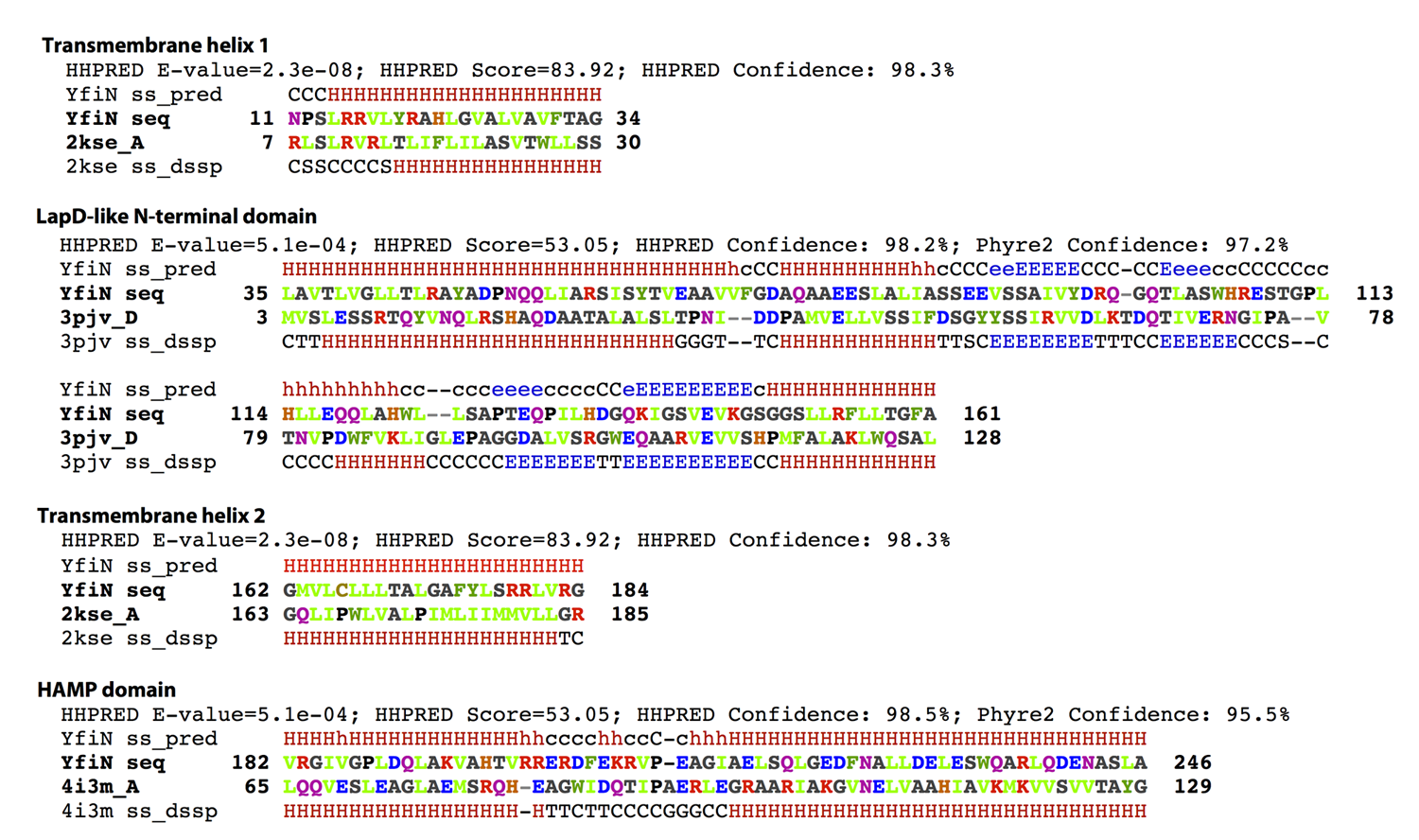

Supplement: Figure S3 — Best templates for homology modelling of full length YfiN. Sequence alignments based on secondary structure prediction of the different domains of YfiN with the most significant structural templates according to two different fold prediction servers (Phyre2 and HHPRED). (TIF) [file pone.0081324.s003.tif]

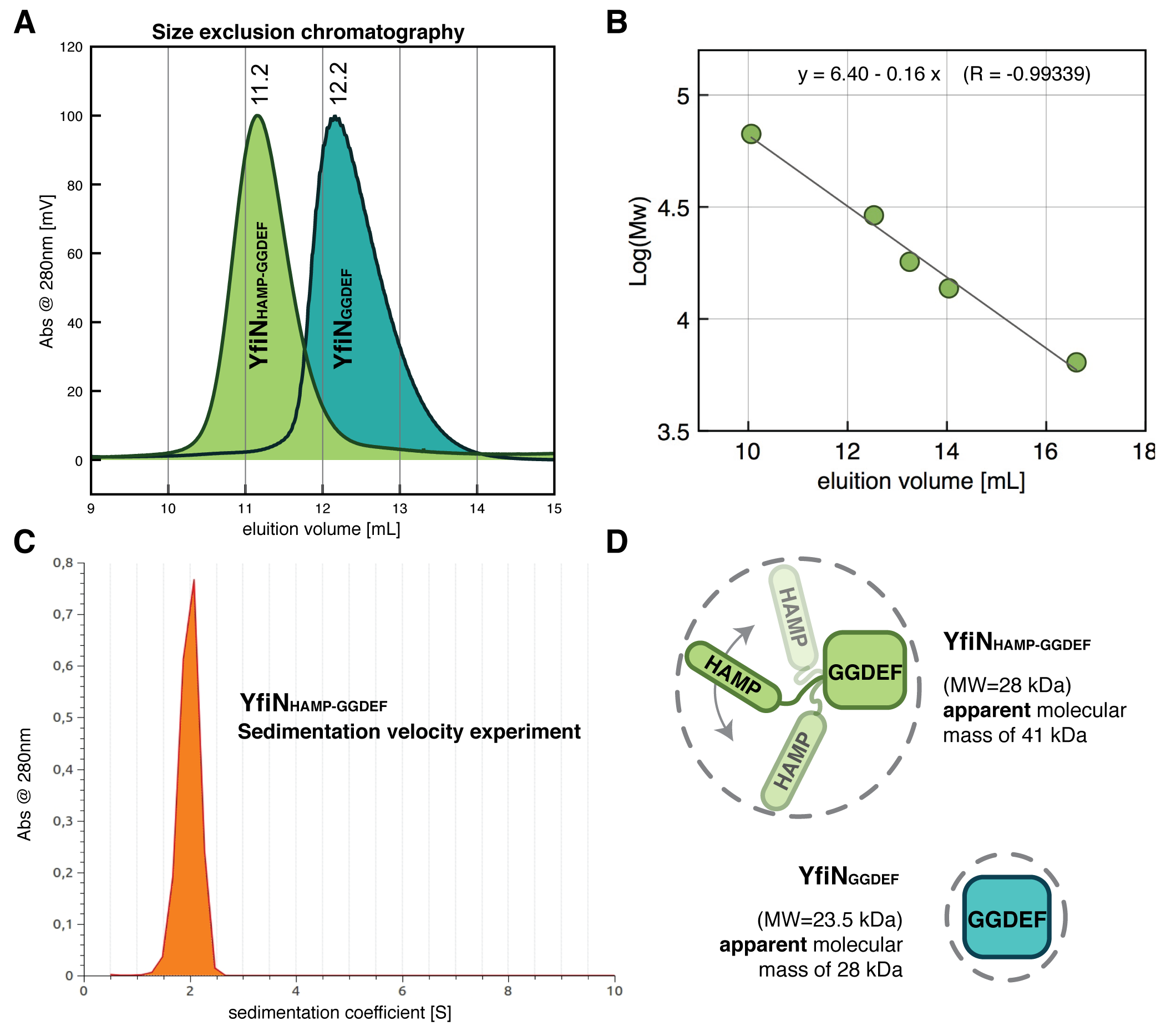

Supplement: Figure S5 — Determination of the aggregation state of YfiNHAMP-GGDEF and YfiNHAMP-GGDEF in solution. A) Size exclusion chromatography (SEC) of YfiNHAMP-GGDEF (green) and YfiNGGDEF (blue) after the affinity chromatography purification step. The proteins elutes with an apparent molecular mass of 41 kDa and 28 kDa respectively. B) Calibration curve obtained using the following standards: BSA 66 kDa; Carbonic Anhydrase 29 kDa; Myoglobin 18 kDa; Ribonuclease A 13.7 kDa and Aprotinin 6.5 kDa. C) Sedimentation velocity experiment to determine the size distribution of YfiNHAMP-GGDEF in solution. The sedimentation coefficient (S) was 2.3 for 98% of the protein, consistent with a molecular mass of 21 kDa, and indicating a monomeric state of YfiNHAMP-GGDEF in solution. D) The YfiNHAMP-GGDEF , the results of the SEC analysis indicates that the two domains of the protein are mobile, thus displaying a large hydrodynamic volume. On the contrary, YfiNGGDEF displays an apparent molecular mass consistent with a monomer, as illustrated in the scheme. (TIF) [file pone.0081324.s005.tif]
