# Supplementary material for: Investigating the Allosteric Regulation of YfiN from Pseudomonas aeruginosa: Clues from the Structure of the Catalytic Domain
Source: PLoS One. 2013 Nov 22;8(11):e81324. doi: 10.1371/journal.pone.0081324 (PMC3838380; doi:10.1371/journal.pone.0081324)
Supplement: Figure S4 — Sequence conservation. Multiple sequence alignment of 53 non-redundant orthologous of YfiN sequences, from other Pseudomonas strains and from more distantly related sequences from other bacteria. (PDF) [file pone.0081324.s004.pdf]

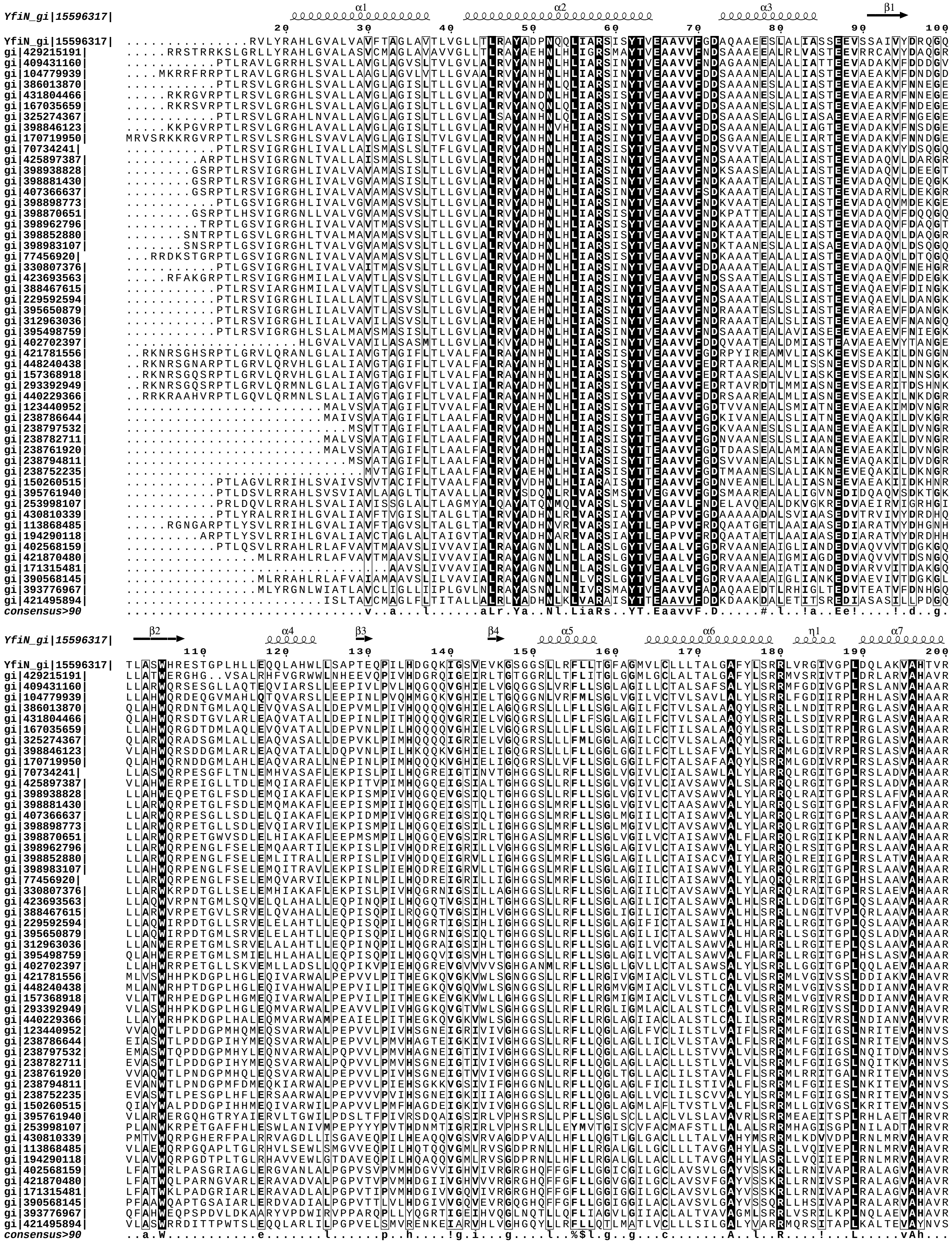
YfiN_gi|15596317|
α1
α2
α3
β1
20
30
40
50
60
70
80
90
100
gi|429215191|
gi|409431160|
gi|104779939|
gi|386013870|
gi|431804466|
gi|167035659|
gi|325274367|
gi|398846123|
gi|170719950|
gi|70734241|
gi|425897387|
gi|398938828|
gi|398881430|
gi|407366637|
gi|398898773|
gi|398870651|
gi|398962796|
gi|398852880|
gi|398983107|
gi|77456920|
gi|330807376|
gi|423693563|
gi|388467615|
gi|229592594|
gi|395650879|
gi|312963036|
gi|395498759|
gi|402702397|
gi|421781556|
gi|448240438|
gi|157368918|
gi|293392949|
gi|440229366|
gi|123440952|
gi|238786644|
gi|238797532|
gi|238782711|
gi|238761920|
gi|238794811|
gi|238752235|
gi|150260515|
gi|395761940|
gi|253998107|
gi|430810339|
gi|113868485|
gi|194290118|
gi|402568159|
gi|421870480|
gi|171315481|
gi|390568145|
gi|393776967|
gi|421495894|
consensus>90
β2
α4
β3
β4
α5
α6
η1
α7
110
120
130
140
150
160
170
180
190
200

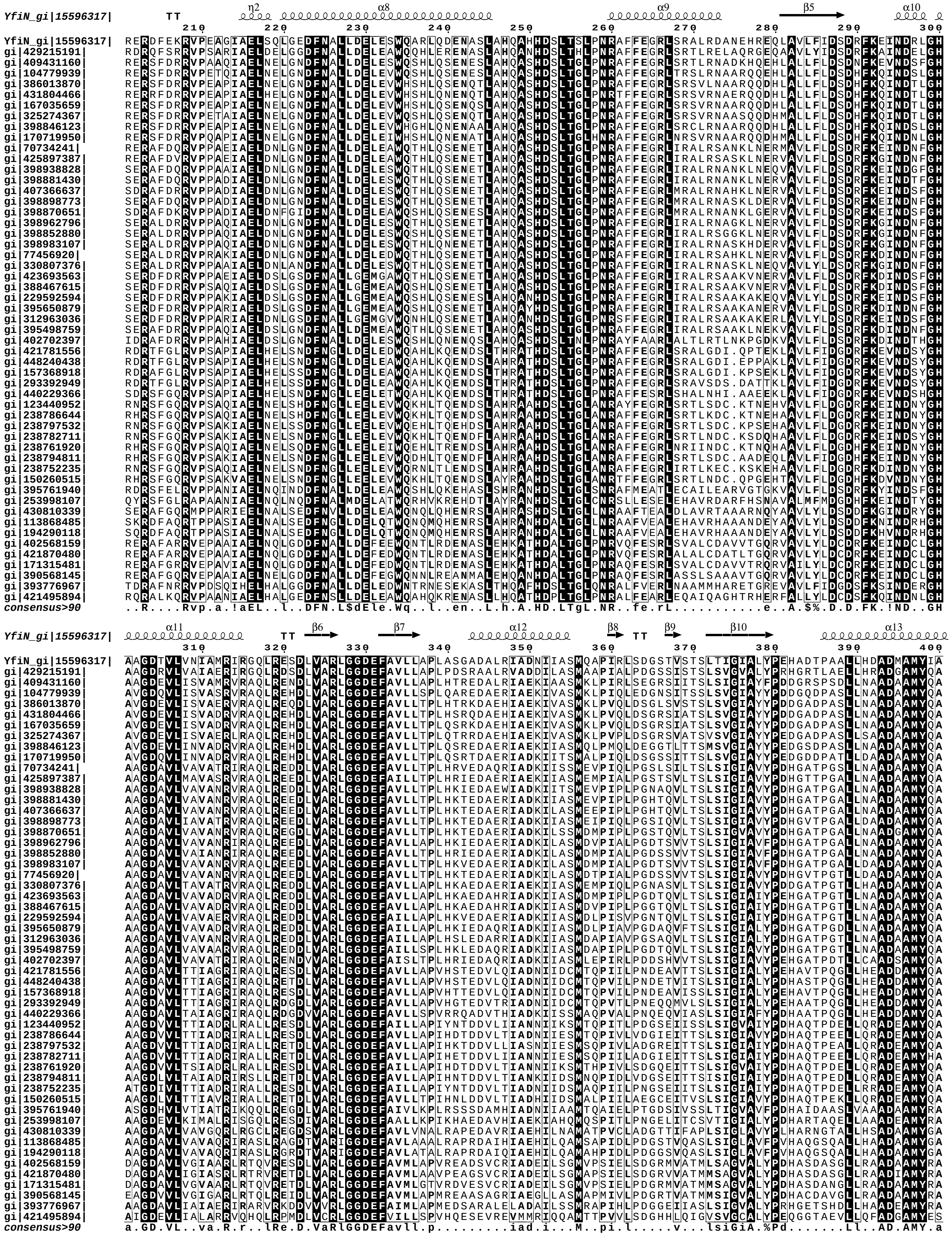

YfiN_gi|15596317|
TT
η2
α8
α9
β5
α10
α11
TT
β6
β7
α12
β8
TT
β9
β10
α13
consensus>90

```
YfiN_gi|15596317|             β11
                              410
YfiN_gi|15596317|  KRQARGSRRLAELND..................
gi|429215191|      KRRGRGMRSLAGEDTDSNTEDYKEIARDSQAPE
gi|409431160|      KRNLRGQGQMAE.....................
gi|104779939|      KRKRRGHWQVAQ.....................
gi|386013870|      KRKRRGHWQVAQ.....................
gi|431804466|      KRKRRGHWQVAQ.....................
gi|167035659|      KRKRRGHWQVAQ.....................
gi|325274367|      KRKRRGHWQVAQ.....................
gi|398846123|      KRKRRGQWQAAQ.....................
gi|170719950|      KRMRRGQWQAAQ.....................
gi|70734241|       KRGSRGGQHTVGAEPPIVQVQN...........
gi|425897387|      KRVSRGGQHTAGSEPPVV...............
gi|398938828|      KRLSRGAQHTAGSEHP.................
gi|398881430|      KRFSRGAQHTSGSEHP.................
gi|407366637|      KRLSRGAQHTSGAEHP.................
gi|398898773|      KRLSRGAQQTAGAEFP.................
gi|398870651|      KRLSRGAQYTAGSEHP.................
gi|398962796|      KRLSRGAQFTAGSESPVV...............
gi|398852880|      KRLSRGAQFTAGSEHLVDP..............
gi|398983107|      KRLSRGAQFTAEAEHP.................
gi|77456920|       KRLSRGAQSTAGSEHP.................
gi|330807376|      KRLARGGQHTAE.....................
gi|423693563|      KRLSSGGQQTAESEHPVV...............
gi|388467615|      KRLSSGGQQTAESEHPVV...............
gi|229592594|      KRLSSGGQQTAESENPVV...............
gi|395650879|      KRLASGGQQTAEPDPP.................
gi|312963036|      KRFAQGGQQTAE.....................
gi|395498759|      KRLSQGGQQTAE.....................
gi|402702397|      KRLARGGQQVAELEYP.................
gi|421781556|      KHRYNGGWRLA......................
gi|448240438|      KHRYNGGWRLA......................
gi|157368918|      KHRYNGGWRLA......................
gi|293392949|      KHRYNGGWRLA......................
gi|440229366|      KHRYHGGWRLA......................
gi|123440952|      KKGFRPCE.QGTAYQSDYIYSENKYPES.....
gi|238786644|      KKGFRPSE.R.......................
gi|238797532|      KKNIQSGGGRVIAHLSD................
gi|238782711|      KKNFQSSGGRAIAQPSD................
gi|238761920|      KKGFR............................
gi|238794811|      KK...............................
gi|238752235|      KK...............................
gi|150260515|      KKNFRSGKRI.......................
gi|395761940|      KQAQRGTRQVARSPQP.................
gi|253998107|      KDQERGS..........................
gi|430810339|      KRQSGATWHSAESAPP.................
gi|113868485|      KRRSGGTWQSAEPRL..................
gi|194290118|      KRRSGGIWQSAEPRL..................
gi|402568159|      KRARPGTWQLAE.....................
gi|421870480|      KRARPGSWQLAE.....................
gi|171315481|      KRTRPGTWQLAE.....................
gi|390568145|      KRARPGSWQFAE.....................
gi|393776967|      KRLGPGSRWLADTGSGLLLSE............
gi|421495894|      KRQRR............................
consensus>90       K................................
```
